# The expression of miRNA encoded by C19MC and miR-371-3 strongly varies among individual placentas but does not differ between spontaneous and induced abortions

**DOI:** 10.1007/s00709-020-01548-3

**Published:** 2020-10-09

**Authors:** Andrea Gottlieb, Inga Flor, Rolf Nimzyk, Lars Burchardt, Burkhard Helmke, Marc Langenbuch, Meike Spiekermann, Susanne Feidicker, Jörn Bullerdiek

**Affiliations:** 1 Center of Human Genetics, University of Bremen, Leobener Strasse 2, 28359 Bremen, Germany; 2 Institute for Pathology, Elbe Clinic Stade-Buxtehude, Bremervörder Strasse 111, 21682 Stade, Germany; 3 Clinic of Gynecology and Obstetrics, Helios Clinic, Altenwalder Chaussee 10, 27474 Cuxhaven, Germany; 4 Department of Gynecology and Obstetrics, Evang. Diakonie-Hospital, Gröpelinger Heerstrasse 406-408, 28239 Bremen, Germany; 5 Department of Obstetrics and Gynecology, University Hospital Frankfurt, Theodor-Stern Kai 7, 60590 Frankfurt am Main, Germany; 6 Institute for Medical Genetics, University of Rostock, University Medicine, Ernst-Heydemann-Strasse 8, 18057 Rostock, Germany

**Keywords:** microRNA, C19MC, miR-371-3, Placenta, Sampling site, Abortion

## Abstract

miRNAs of the largest human miRNA gene cluster at all, i.e., C19MC, are almost exclusively expressed in the placenta. Nevertheless, only little is known about the interindividual variation of their expression and even about possible influence of gestational age, conflicting data is reported as well as for miRNAs of the much smaller miR-371-3 cluster. Our present study aims at the analyses of the expression of miRNAs from both clusters at different times of pregnancy, possible differences between placenta samples obtained from spontaneous or induced abortions in the first trimester, and the possible variation of miRNA expression at different sites within same placentas. miR-371a-3p, miR-372-3p, miR-373-3p, miR-517a-3p, and miR-520c-3p were quantified in 85 samples and miR-371a-3p was quantified in maternal serum samples taken immediately before delivery. While for miRNA-517a-3p and miR-520c-3p the expression increased with increasing gestational age, the present study revealed strong interindividual differences in the expression of miR-371-3 in full-term placental tissue as well as for miRNAs of the C19MC cluster, where the levels differed to a much lesser extent than for the former microRNAs. Also, strong interindividual differences were noted between the serum samples but differences related to the site of the placenta where the sample has been taken from were excluded. For neither of the data from placental tissue, the study revealed differences between the spontaneous and induced abortion group. Thus, the differences do not in general seem to be related to first trimester abortion. It remains to be elucidated whether or not they affect other prenatal processes.

**Electronic supplementary material:**

The online version of this article (10.1007/s00709-020-01548-3) contains supplementary material, which is available to authorized users.

## Introduction

Chromosome 19 microRNA cluster (C19MC) is primate specific and encodes more than 50 mature miRNAs (Bentwich et al. 2005; Bortolin-Cavaillé et al. 2009). As to its presumed evolution, this large cluster has emerged in a relatively short period of time from a preexisting much smaller cluster (miR-371-3) orthologous of which can be detected in all mammalian species (e.g., mmu-mir-290-295 in mice and rno-mir-290-295 in rat) (miRBase 2014; Zhang et al. 2008). Compared with the size of C19MC, little is known about its function. Accordingly, the functions of the corresponding human predecessor cluster miR-371-3 encoding only six mature miRNAs have been elucidated not fully so far. The expression of both clusters is limited to only a few tissues mainly of the embryonic and fetal period of life and C19MC. Apparently, with few exceptions, its expression is almost exclusively restricted to a few extraembryonic tissues as in particular the placenta. In line with these findings, abundant expression of miRNAs of this latter cluster can be noted in tumors derived from these tissues and cell lines derived thereof, respectively (Morales-Prieto et al. 2012), (Strub et al. 2016). In contrast, transcriptional re-awakening in thyroid adenomas seems to be due to rearrangements of transcriptional activators juxtaposed to the gene cluster (Rippe et al. 2010). Formerly, it was thought that its expression is even restricted to the trophoblast but then C19MC-derived miRNAs were also detected in the mesenchymal core of chorionic villi and in the mesenchyme of the amniotic membrane (Flor et al. 2012). miRNAs of this cluster constitute the major part of the miRNA cargo of placental exosomes suggesting that they serve important functions not only in their cells of origin but also in recipient cells of the released exosomes as in particular maternal NK cells (Donker et al. 2012; Ishida et al. 2015; Kambe et al. 2014). One important mechanism seems to be influencing the immune response against viral infections as demonstrated by Delorme-Axford et al. (2013) in a series of experiments. The authors found that placental trophoblast cells are highly resistant to several virus infections and additionally confer their resistance to other nonplacental cells via exosomes containing, among others, specific miRNAs of C19MC. Furthermore, immunomodulatory functions related to the establishment and maintenance of embryo-maternal tolerance have also been assumed to be influenced by these miRNAs (Bullerdiek and Flor 2012; Ishida et al. 2015; Kambe et al. 2014). In addition, there is an increasing evidence that contribution to proper implantation and placenta development is another group of functions addressed by the miRNAs of both clusters (Xie et al. 2014). Accordingly, a comparison of the placental miRNAome between human and macaque revealed conserved expression of miRNAs of the C19MC and miR-371-3 clusters, thus indicating similar functions of miRNAs of both clusters for placentation in nonhuman primates as well (Schmidt et al. 2018). Nevertheless, given the variety of miRNAs encoded by C19MC alone as well as the differences of the seed sequences, it is tempting to assume that the majority of functions of these miRNAs still remain to be fully elucidated (for review, see Malnou et al. 2019). Furthermore, conflicting data exists as for their expression at different time points during pregnancy (Gu et al. 2013; Morales-Prieto et al. 2012). On the other hand, the question if this may be due to different sampling procedures and the position of the sample within the placenta has not been addressed in detail. Therefore, our present study aims at the analyses of the expression of representative miRNAs from both clusters at different times of pregnancy, possible differences between placenta samples obtained from spontaneous or induced abortions in the first trimester, and the possible variation of miRNA expression at different sites within the same placentas. We feel that such study is still missing and its results may help to understand the functions of these miRNAs.

## Materials and methods

### Tissue samples

To assure comparability of the results, all investigations were performed on formalin-fixed paraffin-embedded (FFPE) samples (see e.g. Noack et al. 2011) and accordingly fresh samples as well were subjected to the FFPE procedure prior to isolation of RNA. Placenta samples of induced and spontaneous abortions (GA week 7–33) were retrieved from the archive and comprised cases from 2010 to 2014. Pathological examinations were performed after hematoxylin and eosin staining of the samples for diagnostic purposes. Full-term placental tissue was collected after timely delivery (estimated date ± 2 weeks). Placental samples of women suffering from preeclampsia were excluded. In case of full-term placentas, a randomly sampled piece of placenta was transferred to 4% buffered formalin immediately after delivery followed by paraffin embedding according to standard techniques.

In addition, six samples of three full-term placentas each were obtained from defined sample sites in two depths (A, B, C: chorion plate; D, E, F about 2 cm closer to basal plate) to prove if the collection site has an influence on expression levels. Amniotic membrane was removed before sampling. The sampling points were A and D, near the umbilical cord; B and E, the middle distance between the umbilical cord and the marginal sinus; and C and F, near the marginal sinus. Samples were transferred to 4% buffered formalin immediately and further processed to FFPE samples.

In total, FFPE tissue samples from 85 placentas were examined. These comprised 23 induced abortions (AR), 39 spontaneous abortions (MSA, including missed abortions), and 23 full-term placentas (TE). Three additional full-term placentas were investigated for the analysis of possible influences of the collection site.

### Serum samples

Serum was collected from women shortly before timely delivery (estimated date ± 2 weeks). Blood was centrifuged at 2700 × g for 10 min within 30 min after collection. Serum aliquots were transferred into cryotubes and immediately stored at − 34 °C for 5 to 30 days. Subsequently, the serum samples were frozen at − 80 °C before further processing.

### RNA isolation

For RNA isolation, six to twelve tissue sections from FFPE samples of 5 μm each were used. Total RNA isolation was performed using the innuPREP Micro RNA Kit (Analytik Jena AG, Jena, Germany) according to the manufacturer’s instructions with the following modifications: lysis of the paraffin sections preceding RNA isolation was conducted using TLS-Lysis Solution and Proteinase K from the innuPREP DNA Micro Kit (Analytik Jena AG) without prior deparaffinization. Sections were incubated for 1 h at 60 °C and 15 min at 80 °C.

For RNA isolation from serum samples 200 μl of serum was thawed on ice. Total RNA was extracted using the miRNeasy Mini Kit (Qiagen, Hilden, Germany) according to the manufacturer’s instructions with one minor modification: 400 μl of the aqueous phase were mixed with 600 μl of ethanol.

### Reverse transcription

To quantify the expression of miR-371a-3p, miR-372-3p, miR-373-3p, miR-517a-3p, and miR-520c-3p in FFPE samples and of miR-371a-3p in serum samples, miRNAs were reverse-transcribed into cDNA using the TaqMan microRNA RT kit (Applied Biosystems, Darmstadt, Germany). Of the total RNA of each FFPE sample, 200 ng were used for reverse transcription, whereas 55 ng of total serum RNA were used. Specific miRNA stem loop primers (Applied Biosystems, Darmstadt, Germany) were used (assay numbers: 371a-3p: 002124; 372-3p:000560; 373-3p: 000561; 517a-3p: 002402; 520c-3p: 002402; RNU6B: 001093, 20a: 000580). For serum samples, a primer pool consisting of 0.75 μl each of stem loop primer miR-371a-3p and miR-20a-3p (as an endogenous control) was used. For each sample, a negative control (− RT) was measured and for each microRNA a non-template control was included. The reactions with a final volume of 15 μl were incubated in a thermal cycler (Biometra TGradient or Biometra Trio-Thermoblock, or, for serum samples, GeneAmp PCR-System 2700 (Applied Biosystems)) for 30 min at 16 °C, 30 min at 42 °C, and 5 min at 85 °C, respectively. Samples were stored at 4 °C for immediate qRT-PCR or at − 20 °C for later processing.

### Pre-amplification of RT products in serum samples

Of miRNA-371a-3p and miRNA-20a-3p assay, 0.75 μl were diluted in a 13.5-μl nuclease-free water. The PCR with a final volume of 50 μl (12.5 μl of this solution, 12.5 μl of RT product, 25 μl TaqMan Universal PCR Master Mix (Applied Biosystems)) was performed at 95 °C for 10 min, followed by 14 cycles of 95 °C for 15 s and 60 °C for 4 min using the GeneAmp PCR-System 2700 (Applied Biosystems). The pre-amplification product was diluted 1:5 in nuclease-free water.

### Real-time PCR

Real-time PCR was performed using the Applied Biosystems sequence detection system 7300 (Applied Biosystems, Darmstadt, Germany) in 96-well microtiter plates with a total volume of 20 μl. Each reaction of FFPE sample cDNA consisted of 7.67-μl nuclease-free water, 10-μl TaqMan Universal Master Mix, 1-μl TaqMan microRNA assay, and 1.33-μl probe. Each reaction of serum cDNA consisted of 9 μl of the pre-amplification product, 10-μl TaqMan Universal PCR Master Mix, and 1-μl TaqMan microRNA assay. For each sample, PCR was performed in triplicate and one negative control was run. Non-template controls of cDNA and PCR of each miRNA were run on every plate. PCR conditions were 50 °C for 2 min, followed by 95 °C for 10 min and 40 (serum) or 50 (FFPE) cycles of 15 s at 95 °C and 1 min at 60 °C.

Relative quantification was performed using the ddCT method (Livak and Schmittgen 2001). For FFPE samples, a thyroid tumor with 19q13 rearrangement known to overexpress C19MC and miR-371-3 (S958) served as a positive control. Another thyroid tumor without 19q13 rearrangement and therefore with low expression in both miRNA clusters (S925) was used as a calibrator as described in Flor et al. (2012). In FFPE samples, RNU6B served as endogenous control for normalization according to Luo et al. (2009), Donker et al. (2012), Flor et al. (2012), Gu et al. (2013), and others. For serum samples, miR-20a served as an endogenous control as suggested by Gillis et al. (2013).

### Statistical analysis

The two-sided Wilcoxon signed-rank test was used to compare averages from two groups. The Kruskal-Wallis test was used when more than two groups were compared. Relationships between two observed or measured amounts were quantified by linear regression. A *p* value of less than 0.05 was considered being significant, a *p* value of less than 0.001 was termed “highly significant.” Statistical calculations were done using the R package, version 3.2.3 (R Core Team 2015).

## Results

### miRNA expression levels differ within a broad range

In eighty-five placentas, the concentration of representative miRNAs of the two neighboring clusters C19MC and miR-371-3 was measured. These were miRNA 371a-3p, 372-3p, 373-3p, 517a-3p, and 520c-3p. The samples belonged to either of three subgroups: 23 were placentas collected after timely delivery (TE) (gestational age (GA) 38–42), 23 were induced abortions (AR) (GA 7–13), and 39 were spontaneous abortions (MSA) (GA 7–33) (S1 Table).

First, we analyzed expression levels in full-term placentas, presenting with only a small variety of their GA. Expression was largely different in the investigated miRNAs with low expression of miR-373-3p (median RQ of full-term placentas = 52.5); middle expression of miR-371a-3p (median RQ of full-term placentas: 2300), miR-372-3p (median RQ of full-term placentas: 2697), and miR-520c-3p (median RQ of full-term placentas: 9182); and extremely high expression of miR-517a-3p (median RQ of full-term placentas: 133,200). All five miRNAs analyzed showed a wide expression range with the largest amplitude from lowest to highest expression in miR-371-3 and smaller amplitudes in miR-517a-3p and miR-520c-3p, respectively (Table 1). As the next step, we analyzed the expression levels in induced and spontaneous abortions. The wide expression range observed in full-term placentas was confirmed in both subgroups of abortions as well (Table 1).

**Table 1:** Relative quantification of miRNA expression (fold change) in subsets of placenta samples

	Type of abortion or delivery			
miRNA	All	AR	MSA	TE
**371a-3p**	*n* = 85	*n* = 23	*n* = 39	n = 23
Median	1521	1774	1193	2300
Mean	2436	2413	1636	3814
SD	3002	3006	1832	4056
Range	52.9–15,980	140.7–14,900	52.9–10,720	111.7–15,980
*n*-fold max/min	302	106	203	143
**372-3p**	*n* = 85	*n* = 23	*n* = 39	*n* = 23
Median	1334	1156	984	2697
Mean	2013	1631	1448	3354
SD	2404	2022	1762	3164
Range	45.2–11,890	92.9–10,020	45.2–10,450	113.4–11,890
*n*-fold max/min	263	108	231	105
**373-3p**	*n* = 84	*n* = 22	*n* = 39	*n* = 23
Median	33.28	37.2	29.8	52.5
Mean	64.6	51.4	35.6	126.4
SD	104	60	35	173
Range	1.8–713.9	3.2–299.7	1.8–218.1	6.3–713.9
*n*-fold max/min	397	93	124	113
**517a-3p**	*n* = 65	*n* = 23	*n* = 19	*n* = 23
Median	52,080	36,800	44,850	133,200
Mean	94,800	39,330	54,910	183,200
SD	115,809	27,281	42,704	154,979
Range	1940–481,300	2250–119,400	1940–160,400	25,790–481,300
*n*-fold max/min	248	53	83	19
**520c-3p**	*n* = 84	*n* = 22	*n* = 39	*n* = 23
Median	4540	4472	3224	9182
Mean	7387	5149	4111	15,080
SD	9260	3045	2596	14,734
Range	285.6–49,960	1664–13,840	285.6–13,060	2453–49,960
*n*-fold max/min	175	8	46	20

AR: induced abortion; MSA: spontaneous abortion; TE: delivery at term + − 2 weeks; SD: standard deviation; *n*-fold max/min: ratio of highest expression divided by lowest expression

### Expression levels do not obviously depend on sampling site

**Fig. 1:**
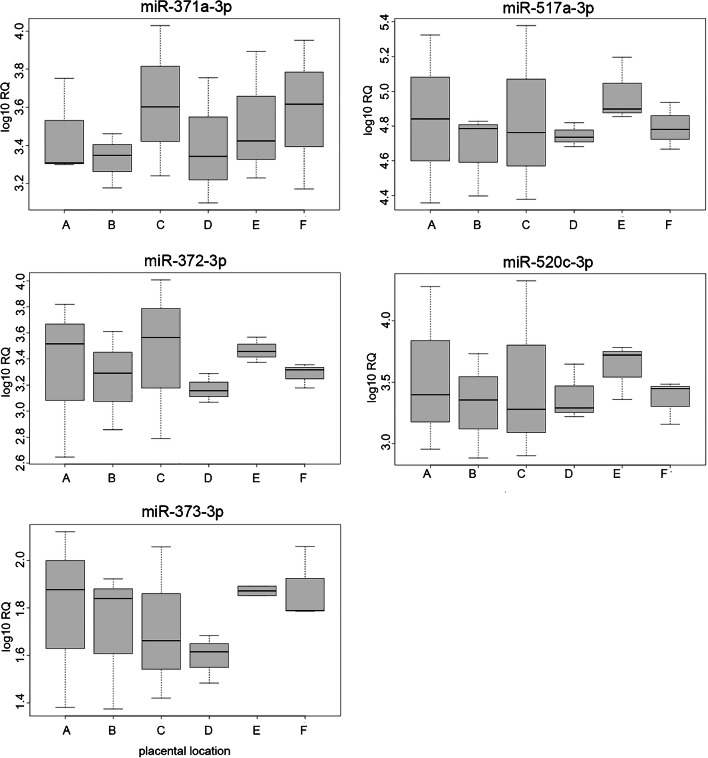
Boxplots showing miRNA expression in six sampling sites of three placentas. A = chorion plate, near umbilical cord; B = chorion plate, middle distance between umbilical cord and marginal sinus; C = chorion plate, near marginal sinus; D = 2 cm closer to basal plate, near umbilical cord; E = 2 cm closer to basal plate, middle distance between umbilical cord and marginal sinus; F = 2 cm closer to basal plate, near marginal sinus. The Kruskal-Wallis analysis of variance showed no significant differences in expression between six sampling sites (miR-371a-3p: *p* = 0.9369, miR-372-3p: *p* = 0.6265, miR-373-3p: *p* = 0.6788, miR-517a-3p: *p* = 0.6159, miR-520c-3p: *p* = 0.8767). Boxes contain the central 50% of values, lines inside boxes denote the median, whiskers extend to the extreme values or to 1.5* box height, whatever is smaller

In our experiments as well as in those described in the literature, the expression level of the miRNAs varied over a broad range and it seemed reasonable to assume that the site where the sample has been taken can influence the results. Thus, to check how the expression varies between different sampling sites and if the broad range in the expression might be determined by sampling site, we have used three additional full-term placentas where six samples each were taken at defined sites. The measurement of all five miRNAs in a total of these 18 samples did not show an obvious influence of the collection site but the individual differences in expression between the three placentas (Fig. 1, S1 Fig.). Accordingly, no differences in expression between means of the six sampling sites and 23 randomly sampled full-term placentas were detected by the Wilcoxon signed-rank test (miR-371a-3p, *p* = 0.7046; miR-372-3p, *p* = 1; miR-373-3p, *p* = 0.7046; miR-517a-3p, *p* = 0.4415; miR-520c-3p, *p* = 0.0785).

### Expression of miR-517a-3p and miR-520c-3p increases significantly with the progression of pregnancy

Concerning the change in expression from first to third trimester, conflicting results have been published. Morales-Prieto et al. (2012) found an increasing expression in miRNAs of the C19MC cluster in the first and third trimester trophoblast cells, whereas no significant changes were observed in miR-371-3. On the other hand, Gu et al. (2013) found a decreasing expression of miR-371-3 and miR-520c and unchanged expression of miR-517a in the first compared with the third trimester placental tissue. To check if and how the expression in the placental tissue changes with progression of pregnancy, we next analyzed the expression patterns of the abovementioned miRNAs as a function of GA. The analysis focused on gestational week without stratification in induced and spontaneous abortion.

**Fig. 2:**
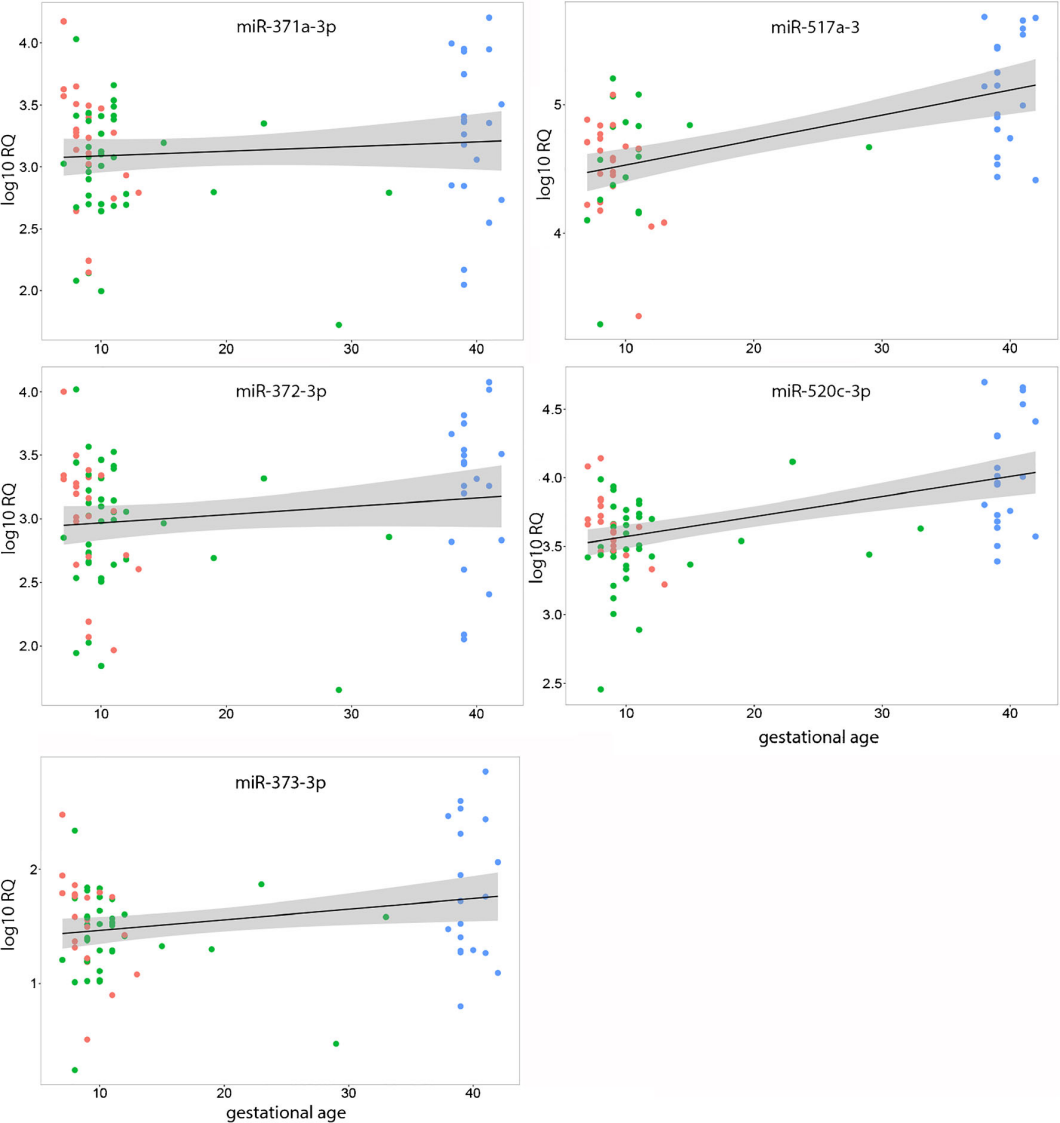
Differences in the expression of placental tissue throughout pregnancy. Linear regression line with 95% confidence range. Red dots, induced abortions; green dots, spontaneous abortions; blue dots, full-term placentas

Due to multiple testing, a Bonferroni correction was made resulting in α = 0.01. Linear regression analysis revealed a highly significant increase from the first to the third trimester of miR-517a-3p (*p* = 0.000003, fold change: 3.9) and miR-520c-3p (*p* = 0.000001, fold change: 3.4). These differences were also noted when comparing samples derived from term placentas with those from induced abortions alone (Fig. 2). In contrast, no significant differences in expression could be observed in different stages of pregnancy in miR-371a-3p (*p* = 0.3905, fold change: 2.0); miR-372-3p (*p* = 0.1474, fold change: 2.2), and miR-373-3p (*p* = 0.018, fold change:3.0) (Fig. 2).

### No differences in expression of induced and spontaneous first trimester abortions were observed

Since miRNAs of the C19MC cluster play a role in viral resistance (Delorme-Axford et al. 2013) and are also suspected to have immunomodulatory functions (Bullerdiek and Flor 2012) and since the miR-371-3 cluster is suspected to be involved in feto-maternal cross talk as well (Rosenbluth et al. 2014; Wang et al. 2016), we were interested to see if variations in the expression of these miRNAs might partly explain miscarriage events. We therefore analyzed the expression levels of induced and spontaneous first trimester abortions separately. The Wilcoxon signed-rank test showed no significant differences in expression of induced compared with spontaneous first trimester abortions in each of the investigated miRNAs (Table 2).

**Table 2:** Expression levels of miRNAs in placental tissue of induced and first trimester spontaneous abortions

miRNA	AR		MSA (first trimester)		*p* value
	*n*	Median RQ ± SD	*n*	Median RQ ± SD	
371a-3p	22	1827 ± 2981	33	1193 ± 1925	0.1734
372-3p	22	1305 ± 2000	33	1060 ± 1855	0.7015
373-3p	22	37 ± 59	33	32 ± 37	0.1963
517a-3p	22	337,610 ± 26,530	19	38,240 ± 44,192	0.7814
520c-3p	21	4567 ± 3008	33	3131 ± 2266	0.1108

Differences were analyzed by the Wilcoxon signed-rank test. A *p* value of < 0.05 was considered significant; a *p* value of < 0.001 was considered highly significant. AR = induced abortion, MSA = spontaneous abortion, RQ = relative quantification, SD = standard deviation

### miRNA concentration of miR-371a-3p in serum samples differs interindividually

The unexpected finding of the wide expression range in placentas prompted us to see if this could be observed in serum as well. While in the literature this variation has been well documented for miRNAs of the C19MC cluster (e.g., Mouillet et al. 2010), less data is available about the presence of miRNAs of the miR-371-3 cluster. Therefore, the concentration of miR-371a-3p, the miRNA showing the widest expression range in full-term placental FFPEs, was measured in four serum samples taken shortly prior to timely delivery. Also in these samples, concentration differed individually, though in a range as half as wide compared with FFPE. The sample displaying the highest CT served as a calibrator. The other three samples showed RQ levels of 14.6, 21.8, and 77.3, respectively.

## Discussion

C19MC is nearly exclusively expressed in the placenta (Bentwich et al. 2005; Luo et al. 2009), suggesting an important role in placental development and function. Specific function and regulation of C19MC in the placenta is not completely clear yet (reviewed by (Mouillet et al. 2015)), but three functional complexes are likely to be influenced by C19MC: these are viral resistance (Delorme-Axford et al. 2013), implantation and placenta development (Anton et al. 2015; Ding et al. 2015; Xie et al. 2014), and immunomodulation in terms of fetal-maternal cross talk (Bullerdiek and Flor 2012; Ishida et al. 2015; Kambe et al. 2014).

This is, to our knowledge, the first report investigating the expression of C19MC miRNAs and miR-371-3 cluster in the placental tissue comparing three groups (induced and spontaneous abortions and full-term placentas) in a large cohort. MiR-517a-3p and miR-520c-3p represented the placenta-specific C19MC. The miR-371-3 cluster was chosen because of its suspected role in stem cell maintenance (Langroudi et al. 2015) and cell differentiation (Kim et al. 2011), pointing at its possible role in trophoblast differentiation.

Overall, individual expression levels differed remarkably within each group (induced and spontaneous abortions and full-term placentas) for each of the five miRNAs investigated. In contrast, Gu et al. (2013) found relatively homogenous expression in six first and six third trimester placental tissues using microarray analysis. Our finding prompted us to examine if expression levels are dependent on sampling site. Analysis revealed that though expression differs within one placenta, it does not show relation to sampling site. Expression levels therefore seem to vary individually. This finding is partly in line with Wyatt et al. (2005) who found no association between placental sampling site and expression of some hypoxia-related genes (NDRG1, adipophilin, and human placental lactogen), whereas others were differently expressed. By investigating the placental transcriptome, Sood et al. (2006) found interindividual differences in the expression patterns of diverse genes as well.

The expression of C19MC, C14MC, and the miR-371-3 cluster was examined by Morales-Prieto et al. (2012) in first and third trimester trophoblast cells. The authors found a tenfold change in miR-520c and about 20-fold increase in miR-517a. Our data confirm this finding, but the increase was lower. Since we investigated placental tissue instead of cell lines, our findings might mirror the in vivo situation more accurately. In concordance with Morales-Prieto et al. (2012), we did not find significant changes in the expression of miR-371-3 cluster throughout pregnancy. An increase of C19MC expression was also observed in the peripheral natural killer cells (NK) of pregnant women in the third compared with the first trimester (Ishida et al. 2015) with a rapid decline post-delivery (Ishida et al. 2015; Kambe et al. 2014). In contrast, Gu et al. (2013) observed a downregulation of miR-371-3 and miR-520c in the placental tissue of third compared with first trimester. We have no straightforward explanation but this conflicting data might partly be due to small sample size used in the latter study. Recently, an evidence for an aberrant expression of miRNAs of C19MC in the third trimester associated with preterm birth has been reported by Hromadnikova et al. (2017).

Xie et al. (2014) found higher expression of C19MC in villous trophoblasts than in extravillous trophoblast, suggesting a role in migration of extravillous trophoblast. The increasing expression in extravillous trophoblast results in the decreasing invasion. Since trophoblast invasion usually takes place during early pregnancy, but can be observed in second trimester pregnancies as well (Pijnenborg et al. 2008), increasing levels of C19MC might restrict invasion as pregnancy progresses and might therefore serve as protection against deep invasion of the trophoblast leading to placenta accreta. According to Umemura et al. (2013), trophoblastic cells of placenta accreta and especially of placenta increta and percreta show aggressive invasion into the myometrium. The authors found miR-34a, a miRNA inversely associated with invasiveness and metastasis, downregulated in placenta accreta. Regulation of trophoblast invasion is a complex process which is tightly regulated by an interaction of diverse factors (reviewed in Lash 2015). For example, the suppression of trophoblast invasion by β-1,4-galactosyltransferase III (B4GALT3) was investigated by Liao et al. (Liao et al. 2015). The authors showed an increased expression of B4GALT3 in third trimester extravillous trophoblasts implicating a role in invasion control. Nevertheless, the potential role of miRNAs of the C19MC cluster in the formation of placenta accreta remains to be elucidated.

A recent study revealed a different expression of miRNAs of the miR-371-3 cluster and C19MC cluster in women suffering from recurrent miscarriage (RM) (Wang et al. 2016). Our hypothesis when starting the study was that miR-371-3 and C19MC might play a role in miscarriage events, but we did not find significant differences in the expression of any of the miRNAs investigated between induced and spontaneous abortions. Wang et al. (2016) focused on women with RM and compared expression in the decidua and in villous trophoblasts. The authors found miR-517a-3p upregulated in the decidua, whereas miR-371a-5p and miR-372 were downregulated in the villi of RM patients compared with induced abortions of normal pregnancies. While the authors did exclude parental chromosomal alterations as a cause of RM, one might speculate if aberrant expression of these miRNAs, e.g., due to paternal deregulation can indicate a general problem of proper placenta development in these couples. From our data, this question cannot be answered because we do not know in which cases repeated abortions had occurred.

Moreover, since we investigated whole FFPE without microdissection and therefore cannot differentiate between the expression of decidua and villi, results might not be comparable. Furthermore, information about RM in our spontaneous abortion group was not available. Nevertheless, taken together, the miRNAs investigated in our study are not likely to play an important role in miscarriage events in general since we did not find significant differences in the expression of induced and spontaneous abortions.

Of note, as to the function of the miRNAs of both clusters, it has to be considered also that they do not only act in the cells where they are expressed but can also traffic between cells, thus exerting their effects in recipient cells. Accordingly, these miRNAs can be detected, e.g., in maternal and fetal venous plasma (Paquette et al. 2018). Luo et al. (2009) first found miRNAs of C19MC in exosomes released from trophoblast cells. Donker et al. (2012) showed that C19MC miRNAs are even predominantly present in these exosomes. These findings suggested a possible use in the monitoring of pregnancy. So far, studies have found that circulating miRNAs of C19MC can indeed serve as biomarkers in preeclampsia, fetal growth restriction, and hypertension (Higashijima et al. 2013; Hromadnikova et al. 2013; Hromadnikova et al. 2014; Kotlabova et al. 2011; Mouillet et al. 2010). Compared with the broad range in the expression of all miRNAs investigated in placental FFPE, individual variation in maternal serum was still high, but nevertheless half as broad. Of note, however, the data presented here is restricted to very few cases and further investigations are required. In the literature, diverse results concerning the correlation of miRNA levels in the placenta and serum have been reported. Higashijima et al. (2013) found different expressions of C19MC miRNAs in the placentas of fetal growth restriction (FGR) compared with normal pregnancies but no differences in serum levels between these groups. On the other hand, Mouillet et al. (2010) found an inverse correlation of placental and serum miRNA levels in FGR pregnancies. Williams et al. (2013) investigated placental tissue and plasma probes of mother-child pairs. Some miRNAs of C19MC were more abundant in maternal and fetal plasma than in the corresponding placenta, and some showed reverse values and others, e.g., miR-517a were highly expressed in the placenta and plasma of both the mother and fetus. However, heterogeneity of serum concentration correlated with the corresponding placenta samples in Williams’ data.

While for the miRNAs of C19MC tested the expression increased with increasing gestational age, the present study revealed strong interindividual differences in the expression of the miR-371-3 cluster in the placental tissue. Differences were also seen for the miRNAs of the C19MC cluster tested, but the levels differed to a much lesser extent than for the former microRNA. As a straightforward explanation, differences related to the site of the placenta where the sample has been taken from were excluded. From our data, these differences do not in general seem to be related to first trimester abortion but it remains to be elucidated whether or not they affect other fundamental processes during prenatal life.

## Electronic supplementary material

ESM 1 (PDF 179 kb)

ESM 2 (XLSX 19 kb)

ESM 3 (DOCX 15 kb)

## Abbreviations

AR: Induced abortions
B4GALT3: β-1,4-Galactosyltransferase III
C19MC: Chromosome 19 microRNA cluster
FFPE: Formalin-fixed paraffin-embedded
GA: Gestational age
MSA: Spontaneous abortions, including missed abortions
RT: Reverse transcription
RM: Recurrent miscarriage
TE: Full-term placentas
