# Supplementary figures and images for: The expression of miRNA encoded by C19MC and miR-371-3 strongly varies among individual placentas but does not differ between spontaneous and induced abortions
Source: Protoplasma. 2020 Oct 9;258(1):209–18. doi: 10.1007/s00709-020-01548-3 (PMC7782366; doi:10.1007/s00709-020-01548-3)

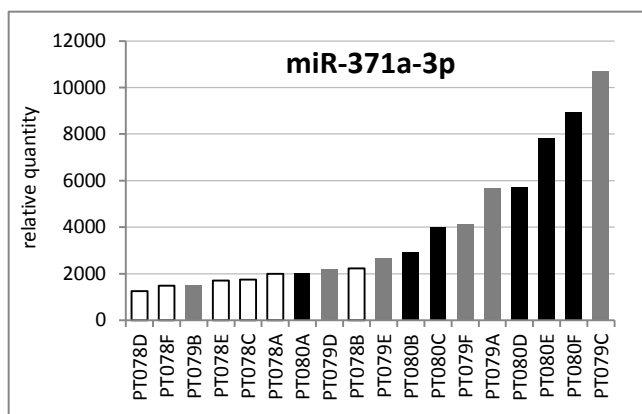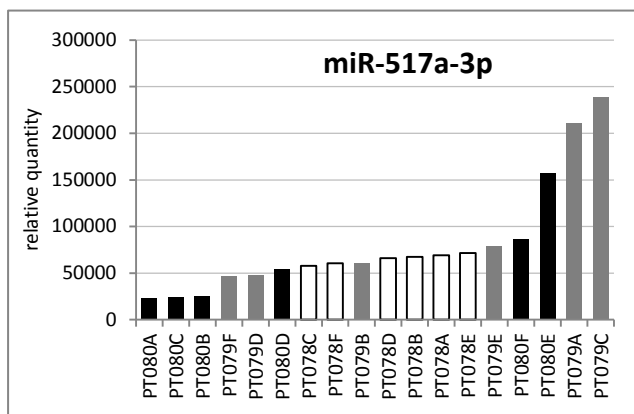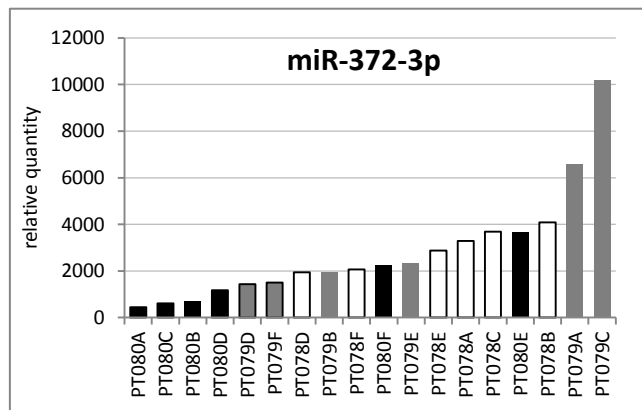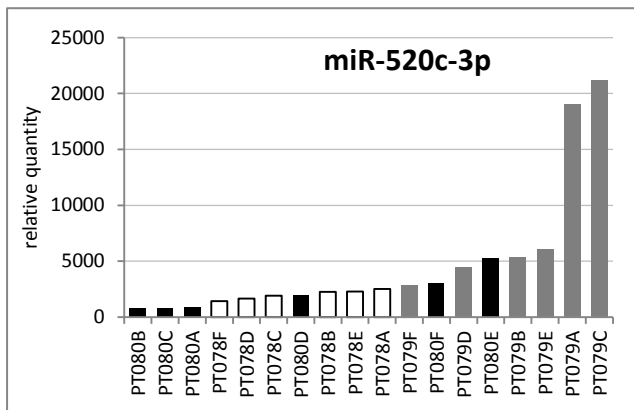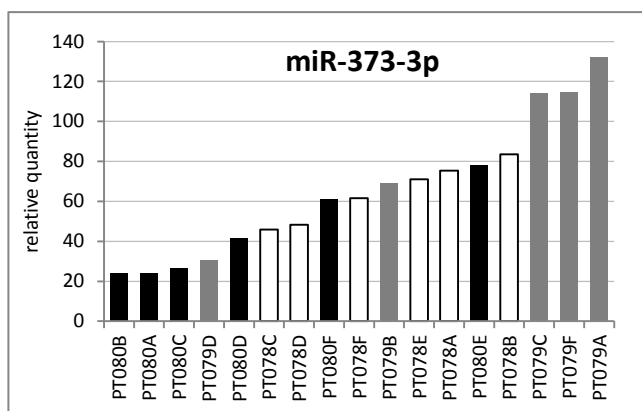

Supplement: Supplementary file 1 — (PDF 179 kb) [file 709_2020_1548_MOESM1_ESM.pdf]
