# Supplementary material for: The expression of miRNA encoded by C19MC and miR-371-3 strongly varies among individual placentas but does not differ between spontaneous and induced abortions
Source: Protoplasma. 2020 Oct 9;258(1):209–18. doi: 10.1007/s00709-020-01548-3 (PMC7782366; doi:10.1007/s00709-020-01548-3)
Supplement: Supplementary file 3 — (DOCX 15 kb) [file 709_2020_1548_MOESM3_ESM.docx]

The expression of miRNA encoded by C19MC and miR-371-3 strongly varies among individual placentas but does not differ between spontaneous and induced abortions

Protoplasma

Andrea Gottlieb^1*^, Inga Flor^1*^, Rolf Nimzyk^1^, Lars Burchardt^1^, Burkhard Helmke^2^, Marc Langenbuch^3^, Meike Spiekermann^1¶^, Susanne Feidicker^4,6^ and Jörn Bullerdiek^1,5^*

Corresponding author: Jörn Bullerdiek, Human Genetics, University of Bremen, Leobener Str. 2, 28359 Bremen, Germany, Email: bullerd@uni-bremen.de; +49 421 218 61500

**Supplementary material**

**S1 Tab.: Overview of all placenta samples used for expression studies**

AR: induced abortion; MSA:spontaneous abortion; TE: delivery at term +-2 weeks; GA: calendar gestastional age in weeks; NA: information not available; Trim: Trimester of pregnancy; RQ: relative quantification; -: not applicable; C section: Caesarean section

**S1 Fig: miRNA expression in six sampling sites of three placentas.**

White columns: samples PT078A-F, grey columns: samples PT079A-F, black columns: samples PT080A-F. A= chorion plate; near umbilical cord, B= chorion plate; middle distance between umbilical cord and marginal sinus, C= chorion plate; near marginal sinus, D= 2 cm closer to basal plate, near umbilical cord, E= 2 cm closer to basal plate, middle distance between umbilical cord and marginal sinus, F= 2 cm closer to basal plate, near marginal sinus.
